# Cardiac Injury in Severe Head Trauma, Incidence and Outcome

**DOI:** 10.1186/2197-425X-3-S1-A486

**Published:** 2015-10-01

**Authors:** A Hasanin, A Kamal, S Mostafa, D Zakaria, R Elsayed, A Mukhtar

**Affiliations:** Cairo University, Anesthesia and Critical Care Medicine, Cairo, Egypt; Cairo University, Clinical and Chemical Pathology, Cairo, Egypt

## Introduction

Although cardiac injury was reported in patients with neurosurgical conditions, few data report cardiac injury in patients with traumatic brain injury (TBI) [[1]].

## Objectives

The aim of this work is to report the incidence of cardiac injury in patients with TBI and its impact on patient outcome.

## Methods

A prospective observational study was conducted on a cohort of 50 patients with severe TBI. Only Patients with isolated severe TBI defined as Glasgow coma scale (GCS) < 8 were included in the study. APACHE II score, GCS, hemodynamic data, serum Troponin I, electrocardiogram (ECG), and echocardiographic examination, and patients' outcome were recorded. a Cardiac Injury Score (CIS) was calculated for all patients (rising troponin = 1, abnormal echocardiography = 1, hypotension = 1), univariate and multivariate analysis for risk factors for mortality was done for all risk factors.

## Results

Among a cohort of 50 patients, age was 31 ± 12, APACHE II was 21 ± 5, and male patients were 45 (90%). Troponin I was elevated in 27(54%) patients, abnormal echocardiography and hypotension were documented in 14(28%) and 16 (32%) patients respectively. the in-hospital mortality was 64%. Risk factors for mortality by univariate analysis were; age, GCS, APACHE II score, serum troponin level, CIS, and hypotension. However, in multivariate analysis the only two independent risk factors for mortality; were APACHE II score (OR = 1.25, 95% confidence interval: 1.02-1.54, P = 0.03) and CIS score

(OR = 8.38, 95% confidence interval: 1.44-48.74, P = 0.018).

## Conclusions

Cardiac injury is common in patients with TBI and associated with increased mortality. the association of high cardiac injury score and poor outcome in these patients warrants further larger study.
